# HIV-1 inhibits IFITM3 expression to promote the infection of megakaryocytes

**DOI:** 10.1093/jmcb/mjae042

**Published:** 2024-10-01

**Authors:** Cyrine Bentaleb, Souad Adrouche, Jade Finkelstein, Christelle Devisme, Nathalie Callens, Claude Capron, Morgane Bomsel, Fernando Real

**Affiliations:** Univ. Lille, CNRS, Inserm, CHU Lille, Institut Pasteur de Lille, U1019 - UMR 9017–CIIL - Center for Infection and Immunity of Lille, F-59000 Lille, France; Univ. Lille, CNRS, Inserm, CHU Lille, Institut Pasteur de Lille, U1019 - UMR 9017–CIIL - Center for Infection and Immunity of Lille, F-59000 Lille, France; Université Paris Cité, CNRS, Inserm, Institut Cochin, F-75014 Paris, France; Univ. Lille, CNRS, Inserm, CHU Lille, Institut Pasteur de Lille, U1019 - UMR 9017–CIIL - Center for Infection and Immunity of Lille, F-59000 Lille, France; Univ. Lille, CNRS, Inserm, CHU Lille, Institut Pasteur de Lille, U1019 - UMR 9017–CIIL - Center for Infection and Immunity of Lille, F-59000 Lille, France; AP-HP, Ambroise Paré Hospital, F-92100 Boulogne-Billancourt, France; Université Paris Saclay, Versailles Saint Quentin-en-Yvelines (UVSQ), F-78047 Guyancourt, France; Université Paris Cité, CNRS, Inserm, Institut Cochin, F-75014 Paris, France; Univ. Lille, CNRS, Inserm, CHU Lille, Institut Pasteur de Lille, U1019 - UMR 9017–CIIL - Center for Infection and Immunity of Lille, F-59000 Lille, France

**Keywords:** HIV-1, megakaryocytes, IFITM3

## Abstract

Despite an undetectable plasma viral load as a result of antiretroviral therapy, HIV-1-infected individuals with poor immune reconstitution harbor infectious HIV-1 within their platelets. Megakaryocytes, as platelet precursors, are the likely cellular origin of these HIV-1-containing platelets. To investigate the mechanisms that allow megakaryocytes to support HIV-1 infection, we established *in vitro* models of viral infection using hematopoietic stem cell-derived megakaryocytes and the megakaryocytic MEG-01 cell line. We observed HIV-1 DNA provirus integration into the megakaryocyte cell genome, self-limiting virus production, and HIV-1 protein and RNA compartmentalization, which are hallmarks of HIV-1 infection in myeloid cells. In addition, following HIV-1 infection of megakaryocyte precursors, the expression of interferon-induced transmembrane protein 3 (IFITM3), an antiviral factor constitutively expressed in megakaryocytes, was inhibited in terminally differentiated HIV-1-infected megakaryocytes. IFITM3 knockdown in MEG-01 cells prior to infection led to enhanced HIV-1 infection, indicating that IFITM3 acts as an HIV-1 restriction factor in megakaryocytes. Together, these findings indicate that megakaryocyte precursors are susceptible to HIV-1 infection, leading to terminally differentiated megakaryocytes harboring virus in a process regulated by IFITM3. Megakaryocytes may thus constitute a neglected HIV-1 reservoir that warrants further study in order to develop improved antiretroviral therapies and to facilitate HIV-1 eradication.

## Introduction

Human immunodeficiency virus/acquired immune deficiency syndrome (HIV/AIDS) chronically affects >39 million people worldwide. It is the prototypical example of an infectious disease caused by a difficult-to-eradicate virus that persists in the lymphoid and myeloid compartments despite combined antiretroviral therapy (cART; Deeks et al., 2021), precluding curative outcomes. Available cART for HIV suppresses viral replication to undetectable levels and restores the quality of life for the majority of people living with HIV. However, 10%–20% of treated people living with HIV do not achieve adequate restoration of their immune status despite adherence to the cART regimen and an undetectable viral load. In these individuals, CD4^+^ T cell blood count remains low (<100–500 cells/µl) even years after cART initiation, indicating immunological failure (Chene et al., 2003; Kaufmann et al., 2003; Baker et al., 2008; Kelley et al., 2009; Yang et al., 2020). Interestingly, we and others have discovered that platelets can harbor infectious HIV-1, despite undetectable residual viremia, potentially promoting viral dissemination to the tissues (Real et al., 2020; Simpson et al., 2020; Baumer et al., 2021). In addition to their hemostatic functions, i.e. the natural process of preventing and stopping bleeding, platelets also modulate immune cell function (Garraud and Cognasse, 2015) and interact with infectious agents such as hepatitis C virus, dengue virus, and HIV (Schrottmaier et al., 2022).

The origins and regulation of HIV-1 acquisition by platelets are poorly understood. Effective antiretroviral therapy suppresses viral replication to undetectable levels, maintaining HIV in a latent state in the form of integrated DNA proviruses (Margolis and Archin, 2017). This mechanism is not applicable to platelets, which are anucleated cell fragments lacking DNA such that they are unable to produce HIV particles from integrated DNA proviruses. In addition, HIV-1 contained within platelets are phylogenetically distinct from latent proviruses detected in circulating CD4^+^ T cells (Real et al., 2020), suggesting that the origin of the viruses harbored by platelets differs from the circulating reservoir. HIV sequestered within platelets is likely produced by infected megakaryocytes, the hematopoietic platelet precursor cells. Megakaryocytes are bone marrow-resident cells that comprise less than 1% of bone marrow cells (Branehog et al., 1975). Infected megakaryocytes may represent an important yet largely overlooked and poorly understood HIV reservoir. Indeed, HIV-1 infection has been demonstrated in megakaryocytes from untreated (Zucker-Franklin and Cao, 1989; Zucker-Franklin et al., 1990; Louache et al., 1991) and cART-treated individuals (Real et al., 2020), although we have observed that detectable HIV-1 proviral DNA and viral RNA are rare in these cells in cART-treated patients (Real et al., 2020). Furthermore, structures similar to virus-containing compartments (VCCs), intracellular structures harboring infectious HIV-1 and a hallmark of HIV infection in myeloid cells (Real et al., 2022; Rodrigues et al., 2022), have been detected in megakaryocytes *in vivo* and *in vitro* at the ultrastructural level (Zucker-Franklin et al., 1990; Boukour et al., 2006), but the mechanism underlying their formation has not been studied due to the lack of a suitable experimental model.

Given their low frequency, obtaining sufficient numbers of megakaryocytes from bone marrow to study the mechanisms of infection and virus-containing platelet production is an extremely challenging task. *In vitro* models are therefore required to investigate the cellular and molecular mechanisms that control productive HIV-1 infection in megakaryocytes.

Terminally differentiated megakaryocytes are short-lived cells (Radley and Haller, 1983; McArthur et al., 2018; Kaushansky, 2021) and cannot sustain long-term HIV-1 infection unless an infected megakaryocyte progenitor is presumed (Sebastian et al., 2017; Renelt et al., 2022). Megakaryocytes are generated in the bone marrow from hematopoietic stem cells (HSCs). During canonical megakaryopoiesis, HSCs differentiate into common myeloid progenitor (CMP) cells, which in turn give rise to megakaryocyte-erythroid progenitors (MEPs), followed by terminal differentiation into megakaryocytes that mature to produce platelets (Machlus and Italiano, 2013). Non-canonical fast-track megakaryopoiesis can occur when CMPs give rise to unipotent megakaryocyte progenitors (MegPs) (Miyawaki et al., 2017). Megakaryocytes and their progenitors possess intrinsic antiviral mechanisms to protect this immune cell lineage from virus-induced injury (Boilard and Flamand, 2019; Campbell et al., 2019). One intrinsic antiviral factor expressed by megakaryocytes and their progenitors is interferon-induced transmembrane protein 3 (IFITM3) (Majdoul and Compton, 2022), which blocks the entry and replication of a variety of viruses (Brass et al., 2009; Weidner et al., 2010; Huang et al., 2011). Here, we investigated whether IFITM3 is implicated in the interaction between HIV-1 and megakaryocytes. We found that HIV-1 infects megakaryocyte precursors and counteracts the role of IFITM3 as a viral restriction factor by downregulating its expression in terminally differentiated megakaryocytes hosting viral components.

Viruses can persist and cause immunological dysfunction in the host by infecting, replicating, and establishing themselves in the tissues where the immune response is triggered, particularly in myeloid and lymphoid immune cells (Mims et al., 2015), which can serve as viral reservoirs. Studies of the mechanisms that control HIV-1 persistence in megakaryocytes contribute to our overall understanding of how the bone marrow may provide a niche for viral persistence in individuals living with HIV/AIDS.

## Results

### Megakaryocyte differentiation in vitro

**Figure 1:**
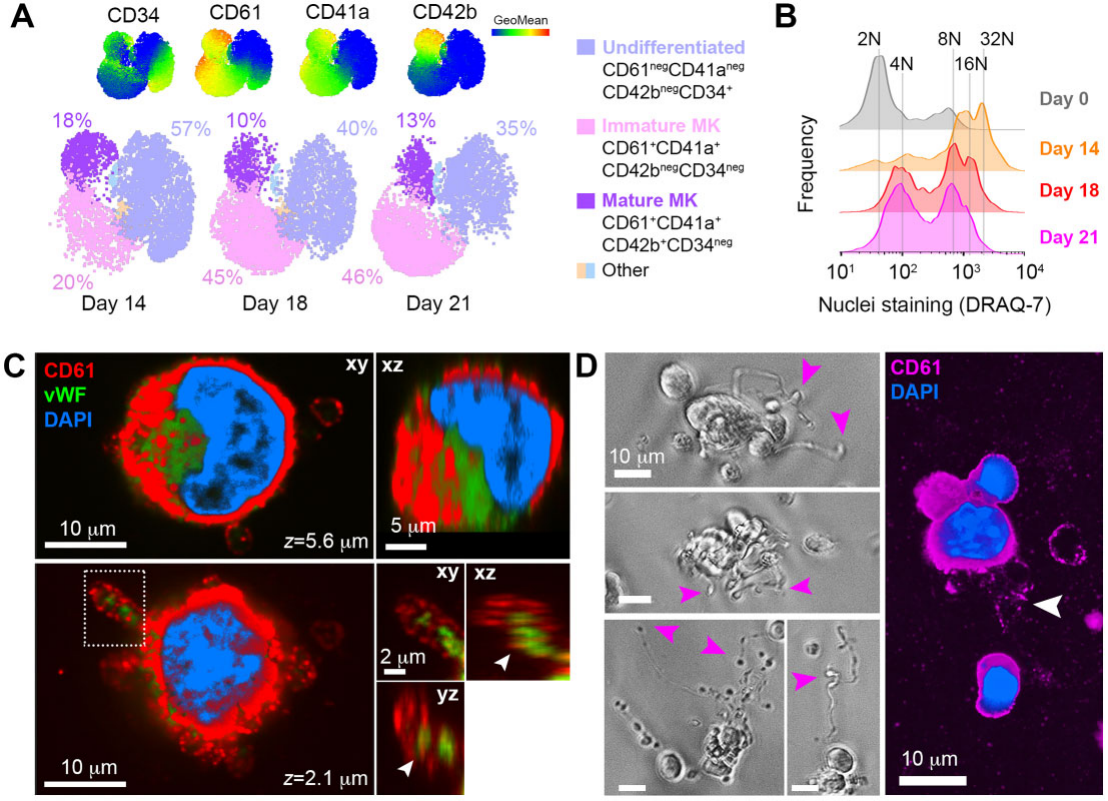
Megakaryocyte differentiation *in vitro*. (**A**) Multiparametric flow cytometry and UMAP analyses of megakaryocytes after 14, 18, and 21 days of differentiation, showing undifferentiated, immature, and mature megakaryocyte populations. (**B**) Frequencies (modal) of megakaryocytes with different ploidy (2N–32N) up to Day 21 of differentiation. (**C**) A CD61^+^ megakaryocyte after 14 days of differentiation with typical megakaryocyte multilobular nuclei. Intracellular maturation marker vWF sequestered in pro-platelets (inset, arrows) was observed as assessed in three-dimensional image xy/xz projections. (**D**) Pro-platelet formation (arrows) in megakaryocyte preparations, observed by phase contrast (left) and confocal microscopy after CD61 immunostaining (right). Results were representative of four different cord blood HSPC donors.

To study the mechanisms controlling HIV-1 infection in megakaryocytes, we used an *in vitro* protocol to differentiate megakaryocytes from primary human cord blood CD34^+^ hematopoietic stem and progenitor cells (HSPCs; Pellin et al., 2019). This protocol resulted in the production of mature CD42b^+^CD41a^+^CD61^+^CD34^neg^ megakaryocytes representing 18.2% (standard error of the mean [SEM] ± 1.6%, *n *= 4) of total cell suspensions after 14 days of differentiation (Figure 1A; Supplementary Figure S1A). Extending the period of *in vitro* differentiation yielded an increase in the frequency of immature CD42b^neg^ megakaryocytes, although the frequency of mature CD42b^+^ megakaryocytes was reduced (Figure 1A). The ploidy of the produced megakaryocytes was used as a parameter to gauge their maturation, revealing that at Day 14 of differentiation, the megakaryocyte population exhibited ploidies ranging from 2N to 32N (Figure 1B). As such, a 14-day differentiation period was selected as the differentiation endpoint. Megakaryocyte maturation was further confirmed by the expression of von Willebrand factor (vWF; Figure 1C) and the detection of CD61^+^ membrane extensions resembling pro-platelet formation, consistent with platelet production competence (Figure 1D).

### Expression of cell receptors and coreceptors for HIV-1 entry in HSPCs

**Figure 2:**
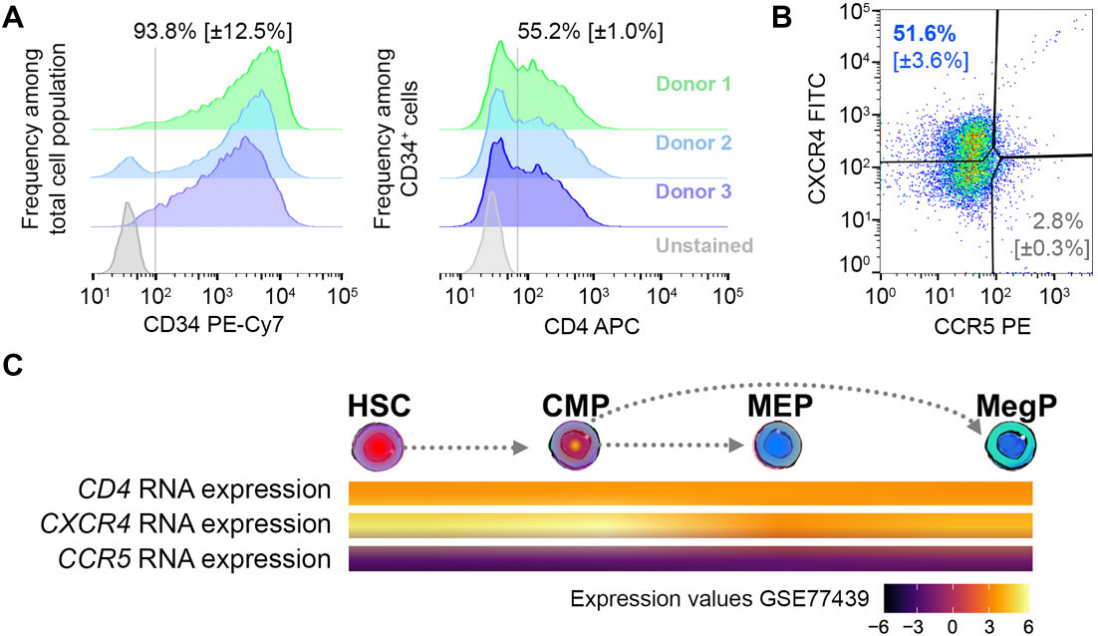
Expression of cell receptors and coreceptors for HIV-1 entry in HSPCs. (**A**) Frequencies (modal) of CD34^+^ cells among total HSPCs in culture (left) and frequencies of CD4^+^ cells among CD34^+^ HSPCs in culture (right). CD4 expression was assessed by flow cytometry (FACS) in HSPCs after HSPC expansion. (**B**) CXCR4 and CCR5 expression in CD4^+^CD34^+^ HSPCs were assessed by flow cytometry at Day 7 of differentiation. Results are representative of three different cord blood HSPC donors. (**C**) mRNA expression levels of *CD4, CXCR4*, and *CCR5* were assessed in HSCs, CMPs, bipotent MEPs, and unipotent MegPs. Results represented as expression values assessed using the GSE77439 dataset. The pathway of HSC differentiation toward the megakaryocyte progenitors MEP and MegP is shown by dotted-line arrows. Expression values represented in colorimetric scale ranging from black–purple (low expression, negative values) to orange–yellow (high expression, positive values) according to the progenitor.

We next set out to develop an approach for studying HIV-1 infection of *in vitro* differentiated megakaryocytes derived from these cord blood CD34^+^ HSPCs. First, we investigated the expression of receptors involved in HIV-1 entry in HSPCs, including the CD4 receptor and the CXCR4 and/or CCR5 coreceptors (Moore et al., 2004). Cord blood CD34^+^ HSPCs exhibited high levels of CD34 expression with a 93.8% (SEM ± 12.5%, *n *= 3) CD34^+^ cell frequency among the overall cell population after HSPC expansion and before the start of the differentiation regimen (Day 0) (Figure 2A; Supplementary Figure S1B). These CD34^+^ cells exhibited high levels of CD4, with 55.2% (SEM ± 1.0%, *n *= 3) of these CD34^+^ cells being positive for CD4 (Figure 2A; Supplementary Figure S1B). These CD4^+^CD34^+^ cells also predominantly expressed the HIV coreceptor CXCR4, with a frequency of 51.6% (SEM ± 3.6%, *n *= 3) CXCR4^+^ cells among CD34^+^CD4^+^ cells, while the HIV coreceptor CCR5 was observed in 2.8% CCR5^+^ cells among CD34^+^CD4^+^ cells (SEM ± 0.3%, *n *= 3) (Figure 2B; Supplementary Figure S1B). These CD4^+^CD34^+^CXCR4^+^ cells were therefore likely to be susceptible to CXCR4-tropic HIV infection.

To assess whether bone marrow megakaryocyte progenitors are susceptible to HIV-1 infection, we performed a survey of a gene expression database (Miyawaki et al., 2017). We evaluated the RNA level expression of the same receptors implicated in viral entry in HSCs, CMPs, MEPs, and the unipotent MegP megakaryocyte progenitors (Miyawaki et al., 2017; Figure 2C; Supplementary Figure S2). The *CD4* mRNA was expressed by all analyzed progenitors at a similar level, while the *CXCR4* coreceptor mRNA levels were higher than those of *CCR5* at all differentiation stages, with increased expression in HSCs and CMPs (Figure 2C; Supplementary Figure S2). We therefore used the CXCR4-tropic HIV-1 strain in the following infection experiments.

### In vitro HIV-1 infection of megakaryocytes

**Figure 3:**
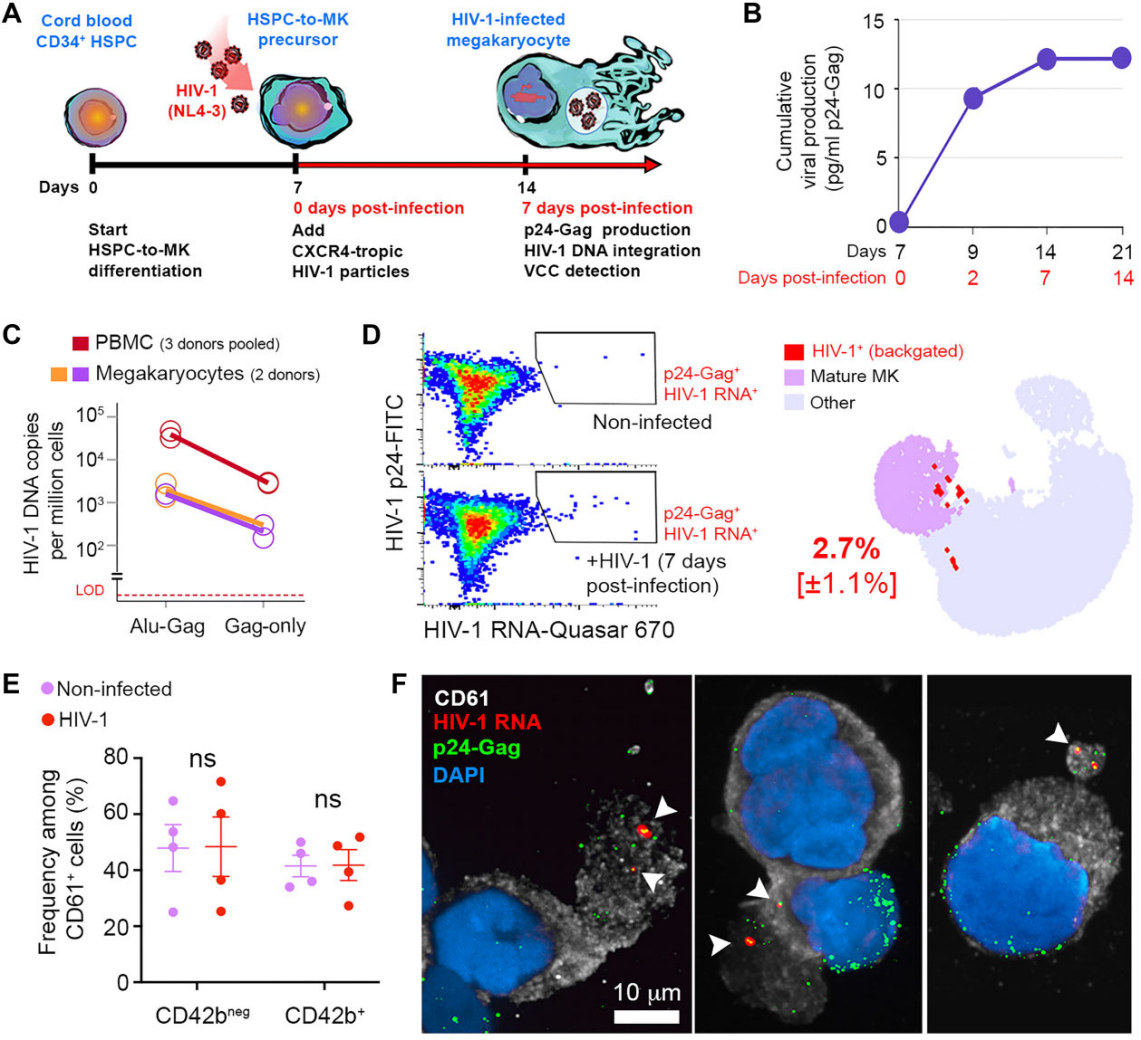
*In vitro* HIV-1 infection of megakaryocytes. (**A**) Experimental design for HIV-1 infection of megakaryocytes. Cells were infected at Day 7 of the differentiation regimen (Day 0 post-infection corresponds to Day 7 of HSPC differentiation). (**B**) Cumulative production of p24-Gag in HSPC culture supernatants over a 14-day period post-infection. (**C**) Integrated HIV-1 DNA copies/10^6^ cells in infected HSPC-derived megakaryocytes. Data show results from two independent donors (orange and purple). A pool of PBMCs (red) from three donors was infected *in vitro* and used as a positive control. Gag-only probing as technical negative control. (**D**) HIV-1 RNA was detected in megakaryocytes by FISH-flow. Left: combined detection of HIV-1 RNA and p24-Gag in megakaryocyte preparations that were or were not infected by HIV-1. Right: p24^+^/HIV-1 RNA^+^ double-positive cells backgated as red dots into megakaryocytes UMAP plots (CD42b^+^ mature megakaryocytes in purple region). The frequency of p24^+^/HIV-1 RNA^+^ megakaryocytes among the mature purple population is presented with SEM in brackets (*n *= 3 donors). (**E**) Frequency of mature (CD42b^+^) and immature (CD42b^neg^) megakaryocytes among CD61^+^ cells infected or not by HIV-1. ns: no statistically significant differences (Mann–Whitney test, *P *> 0.05, *n* = 4 different cord blood HSPC donors). (**F**) Confocal microscopy after HIV-1 RNA *in situ* hybridization combined with immunostaining for HIV-1 p24-Gag protein and megakaryocyte marker CD61, revealing viral compartmentalization in VCC-like structures (arrows).

HIV-1 infection with the CXCR4-tropic strain was performed on Day 7 of the 14-day megakaryocyte differentiation regimen (Figure 3A), in line with what has been reported previously (Voulgaropoulou et al., 2000). As HIV DNA integration and self-limiting production of virions that are stored in VCCs are hallmarks of viral infection in myeloid cells (Real et al., 2018; Ganor et al., 2019), we investigated whether these features were also present in infected megakaryocytes. First, to measure viral release from infected cells, p24-Gag production was assessed in culture supernatants in the days following infection. Viral production increased until 7 days post-infection (Day 14 of differentiation) and reached a plateau from 7 post-infection onwards, indicating a self-limiting production of this viral protein during the process of HSPC-to-megakaryocyte differentiation (Figure 3B). When a post-infection chase period was carried out in medium containing 10 μM of the reverse transcriptase inhibitor Zidovudine (AZT), we observed a 28% (SEM ± 1.5%, *n *= 3) decrease in the output of extracellular p24-Gag in infected HSPC-derived megakaryocyte cultures treated with AZT as compared with untreated cultures (Supplementary Figure S3C and D), demonstrating reverse transcriptase activity. In addition, integrated HIV-1 DNA was detected by nested polymerase chain reaction (PCR; Ganor et al., 2019) in the genome of the total cell population at the 14-day endpoint of megakaryocyte differentiation corresponding to 7 days post-infection (Figure 3C).

Multiparametric flow cytometry combining viral capsid p24-Gag immunodetection and HIV-1 RNA *in situ* hybridization (FISH-flow) (Real et al., 2020) revealed that, at Day 7 post-infection, 2.7% (SEM ± 1.1%, *n *= 3) of mature megakaryocytes contained viral components (p24^+^/HIV-1 RNA^+^) (Figure 3D; Supplementary Figure S3). Importantly, HIV-1 infection did not impact megakaryocyte maturation, as there was no observed effect on the frequency of CD42b^+^ in CD61^+^ cells due to infection (Figure 3E; Supplementary Figure S3B). We then examined the formation of VCC-like structures by confocal microscopy. The HIV-1 capsid protein p24-Gag was found to colocalize with HIV-1 RNA within differentiated megakaryocytes, suggesting viral compartmentalization within VCCs (Figure 3F; Supplementary Figure S4A), similar to what is observed in HIV-1-infected macrophages (Gaudin et al., 2013; Real et al., 2022; Rodrigues et al., 2022). Altogether, these results demonstrate that megakaryocyte precursors can be productively infected by HIV-1, conserving viral components when these precursors mature into terminally differentiated megakaryocytes.

### HIV-1 infection decreases the expression of IFITM3 in megakaryocytes in vitro

**Figure 4:**
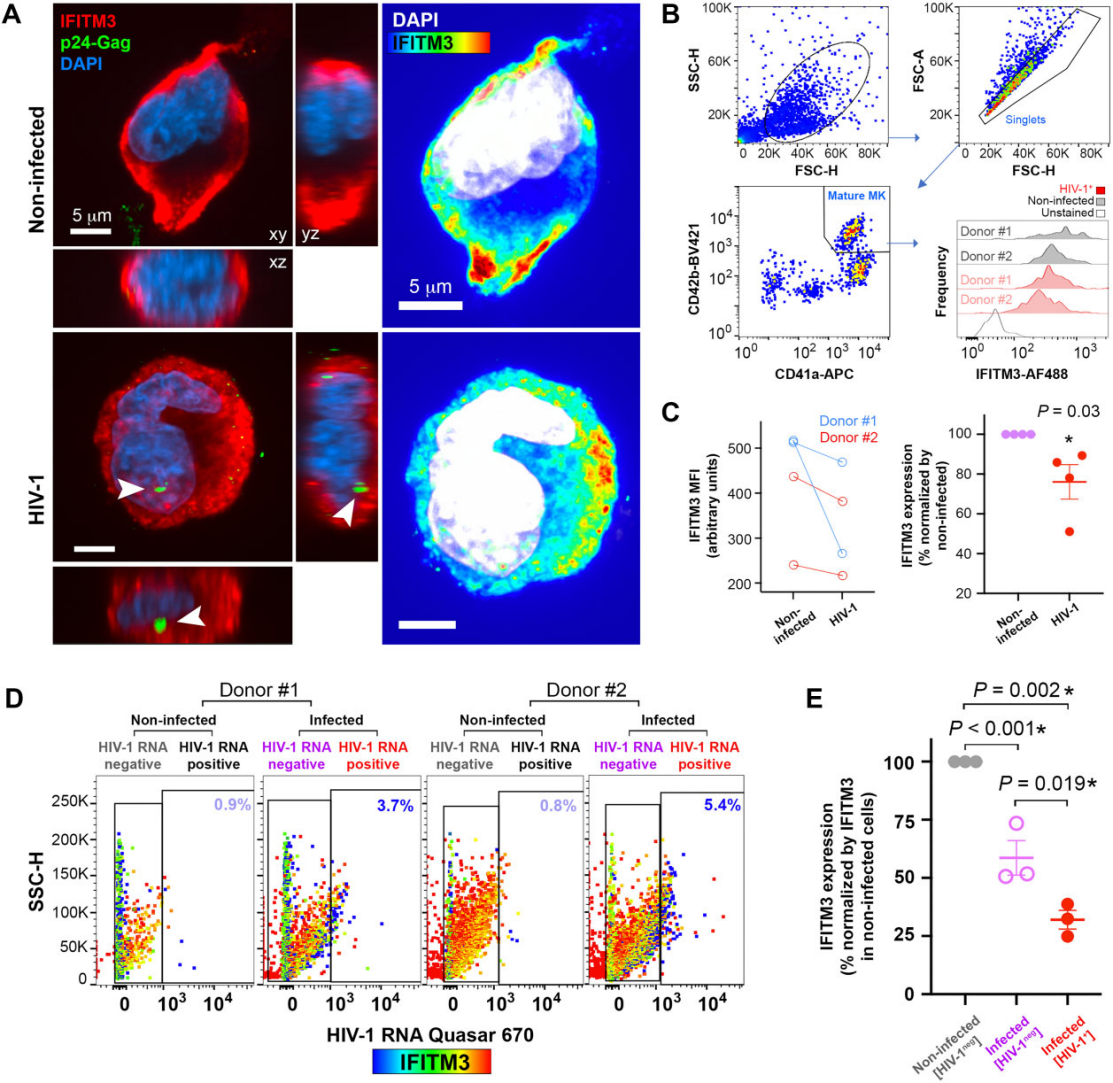
HIV-1 infection decreases the expression of IFITM3 in megakaryocytes *in vitro.* (**A**) Non-infected (upper) and HIV-1-infected megakaryocytes (bottom) were immunostained for HIV-1 p24-Gag (arrowheads) and IFITM3 and observed in three-dimensional xy/xz/yz projections. Nuclei were stained with DAPI. IFITM3 fluorescence intensity represented by colorimetric scale (from blue to red as expression increases), with nuclei (DAPI) in white (Right). (**B**) FACS gating strategy to assess IFITM3 expression in megakaryocytes derived from two different cord blood donors, infected (red histograms) or not (gray histograms) by HIV-1 (7 days post-infection). (**C**) IFITM3 expression (GeoMean MFI) in megakaryocytes infected or not, per HSPCs donor (left), and as percentage of decrease normalized by the non-infected group (right, Mann–Whitney test, *P < 0.05). Results were representative of two different cord blood HSPC donors and two independent experiments. (**D** and **E**) IFITM3 GeoMean MFI in non-infected samples (HIV-1 RNA^neg^ population) and HIV-1-infected samples (HIV-1 RNA^+^ or HIV-1 RNA^neg^ populations) at Day 7 post-infection. HIV-1 RNA^+^ or HIV-1 RNA^neg^ populations were defined by FISH-flow staining of HIV-1 RNA. IFITM3 GeoMean MFI in colorimetric scale (from blue to red as expression increases). Results representative of one experiment using HSPCs from two donors. (**E**) IFITM3 expression levels in HIV-1 RNA^+^ or HIV-1 RNA^neg^ gated populations from HIV-infected cell cultures normalized to the expression levels in the HIV-1 RNA^neg^ gated population from non-infected cell cultures. ANOVA, *P < 0.05. Results were representative of two independent experiments.

Hematopoietic progenitors encode intrinsic antiviral restriction factors that can help preserve hematopoietic lineages and homeostasis (Wu et al., 2018), and the defensive effects of these factors must be overcome by replicating viruses to enable productive infection. One of the viral restriction factors potentially modulated by viral infection is IFITM3, a factor that functions at the convergence between megakaryocyte differentiation (Campbell et al., 2019) and antiviral responses, including responses against HIV-1 (Lu et al., 2011; Compton et al., 2014, 2016; Shi et al., 2017). We evaluated IFITM3 protein expression in mature megakaryocytes in the presence or absence of HIV-1 infection. At Day 14 of differentiation and Day 7 post-infection of cord blood CD34^+^ HSPC-derived megakaryocytes, IFITM3 expression levels were lower in infected cells relative to non-infected mature megakaryocytes, as assessed by both confocal microscopy and flow cytometry. IFITM3 presented no discernible association with the compartmentalized HIV-1 p24-Gag signal (Figure 4A; Supplementary Figure S4B). The virus-induced decrease in IFITM3 expression did not affect the frequency of IFITM3^+^ cells but instead influenced the level of IFITM3 expression as examined by mean fluorescence intensity (MFI). Infected mature megakaryocytes presented a 23.92% (SEM ± 8.65%, *n *= 4) decrease in IFITM3 expression as compared to their non-infected counterparts (Figure 4B and C). In HSPC-derived megakaryocyte cultures infected for 7 days, HIV-1 RNA^+^ cells among the total cell population expressed lower levels of IFITM3 as compared with HIV-1 RNA^neg^ cells and non-infected control cultures (Figure 4D and E; Supplementary Figure S4C). HIV-1 RNA^neg^ cells in infected samples also displayed lower levels of IFITM3 expression as compared with cells from non-infected samples. This indicates that virus-induced IFITM3 downregulation affects both infected and bystander non-infected cells.

### IFITM3 knockdown enhances HIV-1 infection in MEG-01 cells

**Figure 5:**
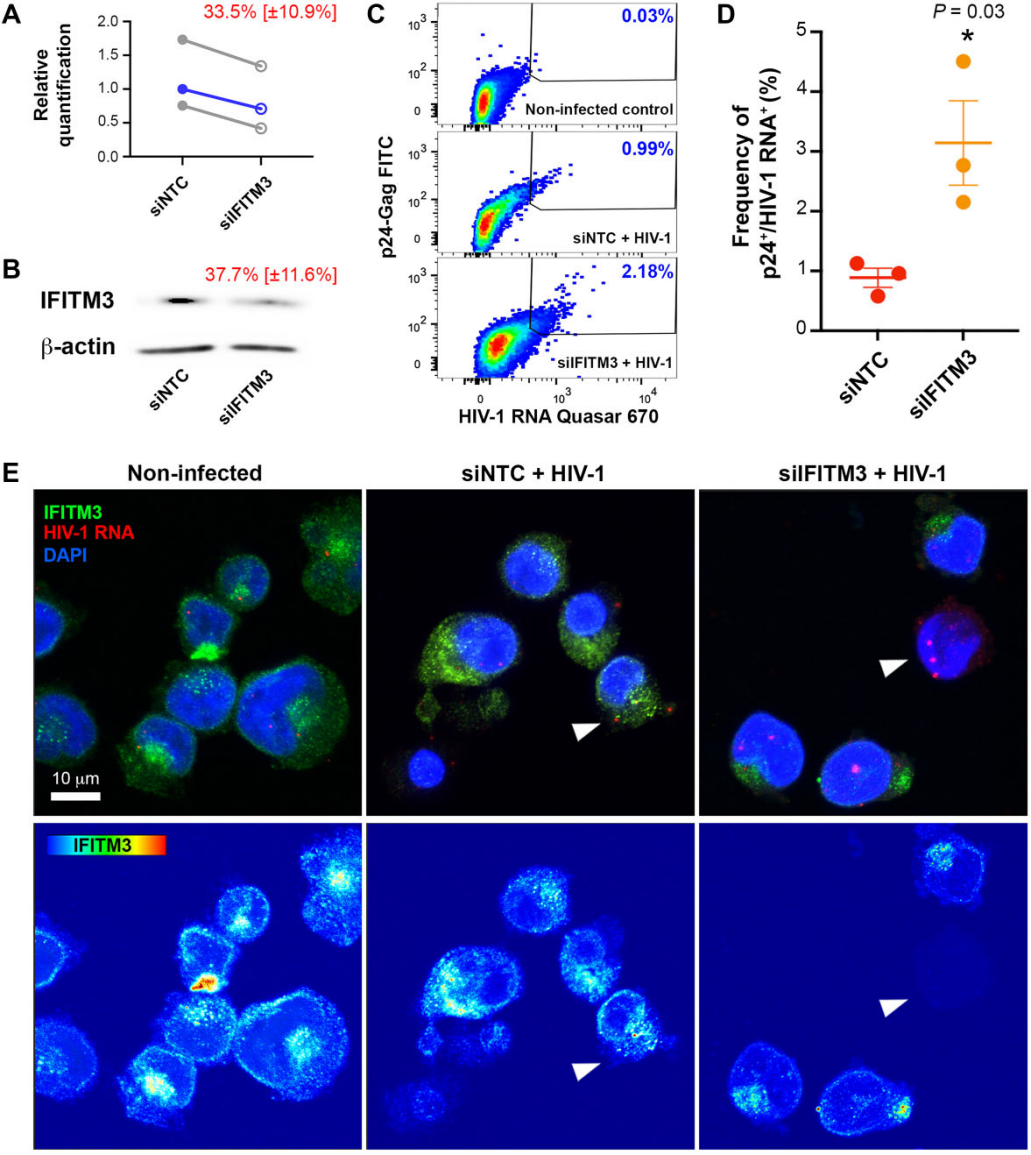
IFITM3 knockdown enhances HIV-1 infection in MEG-01 cells. (**A** and **B**) Relative IFITM3 expression measured in MEG-01 cells transfected with an siRNA targeting IFITM3 (siIFITM3) or a non-targeting control siRNA (siNTC) 48 h post-transfection. The mean percentage (±SEM) of silencing efficiency is shown in red. (**A**) IFITM3 mRNA expression fold change in normalized to RNaseP expression. Blue line indicates mean of independent experiments (gray lines), normalized to siNTC. (**B**) Western blotting of protein lysates from siNTC- and siIFITM3-transfected cells probed with anti-IFITM3 and β-actin antibodies. (**C**) p24^+^/HIV-1 RNA^+^ cell frequency detected by FISH-flow in cells for non-infected control (top), infected siNTC-transfected (middle) and infected siIFITM3-transfected (bottom) cells. (**D**) Frequency of p24^+^/HIV-1 RNA^+^ cells among siNTC- and siIFITM3-transfected infected cells, after subtracting the frequency of p24^+^/HIV-1 RNA^+^ events in non-infected control cultures. Each dot represents an independent experiment (*n *= 3). Student's *t*-test, *P < 0.05. (**E**) HIV-1 RNA *in situ* hybridization coupled to the immunolabeling of IFITM3 in non-infected control (left), infected siNTC-transfected (middle), and infected siIFITM3-transfected cells (right), as observed by confocal microscopy. Arrows point to infected cells.

To demonstrate the direct role of IFITM3 in mature megakaryocyte infection, we knocked down its expression in the megakaryocytic MEG-01 cell line with an IFITM3-specific small interfering RNA (siRNA) prior to HIV-1 infection. The knockdown of IFITM3 resulted in a ∼30% reduction in IFITM3 expression levels, as assessed by quantitative reverse transcription polymerase chain reaction (RT-qPCR) and western blotting (Figure 5A and B). IFITM3 knockdown did not affect IFITM2 expression (Supplementary Figure S5A) and was transient, with peak knockdown at 3 days post-siRNA treatment (Supplementary Figure S5B). *In situ* hybridization coupled with flow cytometry revealed that the frequency of p24^+^/HIV-1 RNA^+^ cells among infected MEG-01 cells increased from 0.9% (SEM ± 0.1%, *n *= 3) to 3.1% (SEM ± 0.7%, *n *= 3) following the knockdown of IFITM3 (Figure 5C and D; Supplementary Figure S5C). These results indicate that the downregulation of IFITM3 favors HIV-1 infection in megakaryocytes. At the morphological level, an increased and compartmentalized HIV-1 RNA *in situ* hybridization signal was observed, consistent with VCC formation (Figure 5E).

## Discussion

Countless studies have emphasized the pivotal role of megakaryocytes in hemostasis. However, these giant bone marrow-derived cells have only recently been acknowledged to exhibit immune functions (Cunin and Nigrovic, 2019). One remarkable aspect of the immune function of megakaryocytes is that they can act as immunomodulators both remotely, via platelets capable of interacting with and immunomodulating immune cells in the peripheral blood, as well centrally in the bone marrow where they act on hematopoietic precursors and immune cell output. Therefore, they may serve as vital—albeit poorly understood—regulators of the immune system and a susceptible target for persistent viruses adapted to overcome host immune defenses.

We here propose that the long-term infection of megakaryocytes by HIV-1 may occur following the early infection of megakaryocyte progenitors residing in the bone marrow. Several viruses can infect HSPCs (Elahi, 2022). HIV-1 can directly infect the HSCs, adversely impacting HSC function and the overall bone marrow stem cell niche (McCune, 2001; Deeks, 2011). Replication-competent HIV-1 proviral DNA has been found in CMPs and MEPs from cART-treated HIV-infected individuals (Sebastian et al., 2017), and it can be transcriptionally active as detectable in mature megakaryocytes (Zucker-Franklin and Cao, 1989; Louache et al., 1991; Real et al., 2020). This suggests that megakaryocyte progenitors can conserve the integrated provirus in their genome and remain infected after maturation, constituting a viral reservoir upon cART treatment and a source of HIV-containing platelets (Real et al., 2020).

In this study, we found that HSPCs are susceptible to CXCR4-tropic HIV-1 infection during their *in vitro* differentiation toward mature megakaryocytes. Infected precursors will generate megakaryocytes exhibiting HIV-1 DNA integration, the combined expression of viral RNA and viral proteins, and the production of viral proteins in megakaryocyte culture supernatants in a self-limiting manner typical of myeloid cell infection. Although the gradual release of viruses stored from the viral input, a key feature of HIV-1 infection in macrophages (Rodrigues et al., 2022) cannot be ruled out, treatment of infected cell cultures with the reverse transcriptase inhibitor AZT reduced extracellular p24-Gag production by ∼30%, collectively suggesting that megakaryocytes can be productively infected when HIV-1 targets megakaryocyte precursors, albeit at a low frequency of cellular infection.

Interestingly, in the HSPC model employed here, the dominant expression of the CXCR4 coreceptor over the CCR5 coreceptor suggests that hematopoietic progenitors may be preferentially infected by CXCR4-tropic HIV, which is linked to disease progression and immune failure in HIV/AIDS (Delobel et al., 2006). Nonetheless, the possibility of the CCR5-tropic HIV infection of progenitors cannot be excluded, as previous studies have revealed the expression of CCR5 in G-CSF/GM-CSF-mobilized CD34^+^ peripheral blood cells and the subsequent CCR5-tropic HIV-1 infection of megakaryocytes derived from these cells (Voulgaropoulou et al., 2000; Renelt et al., 2022).

We have primarily assessed the hypothesis of whether HIV-1 can directly infect megakaryocyte precursors, and whether the virus is conserved within terminally differentiated megakaryocytes. After demonstrating our primary hypothesis, we extended our study to the secondary goal of clarifying the mechanism underlying durable HIV-1/megakaryocyte interactions in an easy-to-manipulate megakaryocyte cell line model system amenable to the silencing of IFITM3 so as to clarify its importance in this context. The HIV-1 infection of megakaryocytes in their progenitor state alters the expression of genes involved in viral response, including IFITM3, allowing for the virus to persist in these cells. We found that the HIV-1 infection of megakaryocyte precursors resulted in the generation of terminally differentiated infected megakaryocytes with diminished IFITM3 expression. HIV-1-mediated IFITM3 downregulation in infected cell cultures was observed in HIV-1 RNA^+^ cells, although the low frequency of infected cells is a limitation to accurately assess this downregulation at the single-cell level. Importantly, despite this low frequency of infected cells, HIV-1-induced inhibition of IFITM3 expression impacted both infected and bystander non-infected cells in this model system. This may be attributable to interactions with viral components and the production of secreted factors in response to these interactions at the cell population level, although further testing will be necessary to explore this hypothesis.

Some limitations of our *in vitro* experimental system should be taken into consideration when considering the impact of HIV-1 in hematopoietic megakaryocyte progenitors and terminally differentiated megakaryocytes in infected individuals. The use of primary cord blood HSPCs for *in vitro* differentiation into megakaryocytes and the *in vitro* HIV-1 infection of megakaryocyte precursors represent important sources of variability in our study. In addition, the siRNA-mediated knockdown of IFITM3 expression was only moderate and transient in the utilized megakaryocytic cell line, limiting our ability to address the participation of IFITM3 in counteracting the initial infection of megakaryocytes while achieving the best balance between knockdown efficiency and cell viability. However, further studies using stable IFITM3 knockout systems are required to address how long IFITM3 downregulation can be maintained in infected megakaryocytes to sustain viral infection in these cells.

IFITM3 plays a crucial role in the innate response against viral infections by hampering viral fusion and endocytosis. It has been shown that IFITM3 inhibits the initial step of infection, namely fusion, for several enveloped viruses such as Influenza A virus (IAV; Desai et al., 2014), murine leukemia virus (Ahi et al., 2020), and SARS-CoV-2 (Xu et al., 2022). IFITM3 also inhibits the fusion of endocytosed viruses with the membrane of late endosomes, a crucial step in the replication of certain viruses like IAV (Feeley et al., 2011). The broad-spectrum antiviral activity of IFITM3 implies that it is an important component of the host defense against a wide range of viral pathogens. For instance, studies of IAV have demonstrated that decreased IFITM3 levels result in increased viral infectivity and replication (Desai et al., 2014). IFITM3 restricts HIV-1 infections at different stages of the HIV-1 replicative cycle by blocking viral entry through IFITM3 incorporation on virions and direct interactions between IFITM3 and HIV-1 *env* (Yu et al., 2015; Drouin et al., 2021), and at later stages of infection when IFITM3 blocks viral protein synthesis (Lee et al., 2018).

Importantly, IFITM3 is an intrinsic antiviral factor (Majdoul and Compton, 2022) expressed by megakaryocytes. It has been demonstrated both *in vivo* and *in vitro* to combat dengue virus (DENV) infection (Campbell et al., 2019). IFITM3 expression is increased in megakaryocytes and platelets during acute dengue and COVID-19 infections (Campbell et al., 2019; Manne et al., 2020; Zhu et al., 2022a), inhibiting, for instance, DENV entry and replication in megakaryocytes (Campbell et al., 2019). However, in contrast to acute *in vitro* DENV infection, in this study, we found that the HIV-1 infection of megakaryocyte precursors at Day 0 post-infection inhibits IFITM3 expression later on at Day 7 post-infection when megakaryocytes are terminally differentiated. This may represent a mechanism that supports latent/persistent infection instead of acute infection. On one hand, HIV-1 inhibits IFITM3 expression in infected, matured megakaryocytes, which may contribute to persistent HIV infection. On the other hand, when assessing the effect of IFITM3 on infection, we found that silencing IFITM3 enhanced HIV-1 infection, indicating that HIV counteracts the intrinsic antiviral response of megakaryocytes mediated by IFITM3.

Although this is the first time, to our knowledge, that IFITM3 downregulation has been demonstrated in HIV-1 infection, Chikungunya virus and Mayaro virus have been reported to induce IFITM3 downregulation in infected cells (Franz et al., 2021). This downregulation could be promoted by viral components that will antagonize the interferon signaling pathways and the expression of interferon-stimulated genes like IFITM3. HIV-1 *nef* can counteract the blockade of HIV-1 protein synthesis imposed by IFITM3 (Lee et al., 2018), while HIV-1 *vif* targets key components of the IFNα JAK/STAT pathway (Gargan et al., 2018) and may be involved in the virus-induced downregulation observed in megakaryocytes. In this study we found that the HIV-1 infection of cord blood CD34^+^ cell-derived megakaryocyte precursors inhibits IFITM3 expression on Day 7 post-infection when megakaryocytes are terminally differentiated *in vitro*. As we observed IFITM3 downregulation in the later stages of HIV-1 infection and not during the acute phase, it is therefore reasonable to speculate that the viral mechanisms controlling IFITM3 expression may differ. Remarkably, but consistent with findings for DENV (Campbell et al., 2019), we found that HIV-1 replication increases significantly by 3 fold when IFITM3 levels are reduced through siRNA-mediated knockdown. Our results substantiate the finding that IFITM3 serves as a robust antiviral factor against HIV-1 in megakaryocytes. Further studies will be required to elucidate the precise mechanism underlying the observed HIV-mediated IFITM3 downregulation in precursors and terminally differentiated megakaryocytes.

The presence of HIV-1 in platelets has harmful effects on the immune system, particularly on CD4^+^ T cells during HIV-1 infection (Zhu et al., 2022b). Due to the infrequent occurrence of infected cells in the myeloid lineage (Real et al., 2018, 2022; Ganor et al., 2019; Veenhuis et al., 2023), investigating the regulatory pathways associated with HIV-containing platelet production in infected megakaryocytes, especially IFITM3 downregulation, poses a challenge. HIV-containing platelets are present in the peripheral bloodstream at frequencies of about 0.1% of total platelets along with platelets lacking HIV (bystander platelets) (Real et al., 2020). It is probable that the majority of platelets formed from infected megakaryocytes do not harbor virions or viral components. The punctate pattern of p24^+^/HIV-1 RNA^+^ is suggestive of virus-containing compartments—as demonstrated in macrophages by p24-Gag immunostaining that was validated by infection with viruses expressing fluorescently tagged Gag and electron microscopy (Gaudin et al., 2013). It should be noted that at the morphological level, patterns consistent with VCCs detected in megakaryocytes derived from cord blood HSPCs were also found in the context of membrane budding (Figure 3E), which bears resemblance to the platelet-generating structures reported by others (Potts et al., 2020). The VCC-like structures, we detected here, in megakaryocytes appear to be smaller than those detected in latently infected macrophages (Real et al., 2018, 2022). This reduced size could allow megakaryocyte VCCs to be stored in platelets during thrombopoiesis.

By exploring the intricate interactions between IFITM3 and different viruses, future research may reveal innovative approaches to modulating IFITM3 expression or function, opening avenues for the development of improved antiviral therapy. This study also contributes to our understanding of the establishment of a persistently infected niche in the bone marrow, wherein megakaryocytes and bone marrow-resident hematopoietic progenitors may participate in the formation of viral reservoirs.

## Materials and methods

### In vitro differentiation and infection of HSPC-derived megakaryocytes

Megakaryocytes were differentiated *in vitro* from CD34^+^ cord blood-derived HSPCs (STEMCELL, #70008.2; Pellin et al., 2019). HSPCs were first expanded in StemSpan SFEM culture medium (STEMCELL, #09600) supplemented with StemSpan CD34^+^ Expansion Supplement (STEMCELL, #02691) as recommended by the manufacturer. An expanded CD34^+^ HSPC frozen cell stock was established with aliquots for single-use being stored in liquid nitrogen. The HSPCs (10^5^ cells) were then thawed and cultivated using StemSpan SFEM culture medium supplemented with StemSpan Megakaryocyte Expansion Supplement (STEMCELL, #02696, containing human stem cell factor, thrombopoietin, IL-6, and IL-9) for 14 days (differentiation protocol) as recommended by the manufacturer. This differentiation protocol was extended up to 21 days according to the selected experimental design. Cultures were replenished with fresh SFEM containing the StemSpan Megakaryocyte Expansion Supplement weekly as recommended by the manufacturer. To assess ploidy, cells were labeled with the double-stranded DNA intercalant DRAQ7 probe after the RNAse treatment of paraformaldehyde-fixed cells as described previously (Raslova et al., 2006).

Cells were infected with CXCR4-tropic HIV-1 on Day 7 after starting the differentiation protocol, when the cells are considered megakaryocyte precursors (HSPC-to-MK). The virus was added to cell cultures at a multiplicity of infection (MOI) of 0.1 (corresponding to 5 ng/ml p24-Gag) for 16 h at 37°C (pulse) and washed out before proceeding through the differentiation protocol until Day 14 (7 days post-infection) and Day 21 (14 days post-infection) (chase). To assess reverse transcriptase activity, cells were cultivated in medium containing 0 or 10 μM AZT (Sigma-Aldrich, A2169) throughout the chase period.

### IFITM3 gene silencing and MEG-01 cell infection

MEG-01 cells (CRL-2021; ATCC) were cultured in RPMI-1640 medium (Gibco) supplemented with 10% heat-inactivated Fetal Bovine Serum (Gibco), 1% Glutamax, and 1 mM sodium pyruvate (Gibco) at 37°C. MEG-01 cells (10^6^ per test) were transfected with siRNA pools (Thermo Fisher Scientific) designed to target IFITM3 (siIFITM3, s195033), IFITM2 (siIFITM2, 215705), or a nontargeting siRNA control (siCTL, Silencer™ Negative Control No. 1 siRNA), using the Neon Transfection System (Thermo Fisher Scientific) as per the manufacturer's instructions. The knockdown of target RNAs was assessed 48 h after transfection by immunofluorescence, western blotting, and RT-qPCR or at 2, 3, and 4 days post-siRNA treatment by flow cytometry. MEG-01 cells were infected with the CXCR4-tropic HIV-1 at an MOI of 0.1 for 16 h at 37°C. Infection of MEG-01 cells was assessed 48 h post-infection. Infection of siRNA-transfected MEG-01 cells was performed at 24 h post-siRNA treatment.

### Flow cytometry and FISH-flow

Cells were fixed with 4% paraformaldehyde in PBS and immunostained with or without combined *in situ* hybridization. Immunostaining of 5 × 10^5^ cells/test was performed with the following antibodies designed and validated for flow cytometry: anti-CD34 PE-Cy7 (1:20; BD, #348811), anti-CD41a BV510 (1:20; BD, #563250), anti-CD61 PE REAfinity (1:20; Miltenyi Biotec, #130-110-749), anti-CD42b BV421 (1:50; BD, #740075), anti-CXCR4 FITC (1:20; R&D, #FAB170F), and anti-CD195 (CCR5) PE (1:20; Pharmingen, #555993) as described previously (Zhu et al., 2022a). IFITM3 indirect intracellular immunostaining was performed using rabbit anti-human IFITIM3 (1:100; Invitrogen, #MA5-32798) for 2 h at room temperature followed by secondary antibody anti-rabbit AF488 (1:200; Invitrogen, #A21206) for additional 2 h at room temperature, applying protocols described previously for other intracellular targets (Zhu et al., 2022a).

For *in situ* hybridization coupled with immunostaining and flow cytometry (FISH-flow), cells were immunostained first, including intracellular staining with anti-p24 (HIV) KC57-FITC (1:20; Beckman Coulter, #6604665) and then processed for *in situ* hybridization as we described previously (Real et al., 2022). Data were acquired with a GUAVA 12HT system (Merck Millipore, Merck, KGaA) or a BD LSR Fortessa flow cytometry system (BD Biosciences). Compensation and fluorescence specificity were established using single-stained and unstained controls.

Flow cytometry data were analyzed with the FlowJo software (FlowJo version 10.9.0, Becton Dickinson & Company). The gating strategy included gating cells based on forward (FSC) and side scatter (SSC) followed by the exclusion of doublets in the FSC-A/FSC-H dot plot. Mature megakaryocytes were retrieved by gating the singlets first on CD61^+^CD34^neg^ populations, then on double-positive CD41a^+^CD42b^+^ populations.

Uniform Manifold Approximation and Projection for Dimension Reduction (UMAP) was generated via the concatenation of HSPC-derived megakaryocyte populations at different time points of the differentiation regimen (14, 18, and 21 days) as described previously (Real et al., 2022), including CD34, CD61, CD41a, and CD42b markers and the FlowJo UMAP plugin. Unsupervised clustering UMAP analyses were performed with the FlowSOM plugin to define mature megakaryocyte populations. Events double-positive for HIV-1 RNA and p24-Gag (p24^+^/HIV-1 RNA^+^) were backgated into the UMAP plot to identify HIV-1 infected populations. Non-infected controls were used to define the fluorescence threshold of HIV-positive cells.

IFITM3 expression was assessed based on MFI GeoMean data as obtained from the FlowJo software in populations gated according to the chosen experiment.

### Immunostaining and in situ hybridization

HSPC-derived megakaryocytes that were or were not infected with CXCR4-tropic HIV-1 were fixed with 4% paraformaldehyde for 20 min at room temperature, washed with PBS, and spotted on Poly-L-Lysine-coated slides to dry as described (Zhu et al., 2022a). For infected and non-infected MEG-01 cells, cells were fixed with 4% paraformaldehyde for 20 min at room temperature, washed with PBS, and spotted on coverslips to dry. Next, the coverslips were glued to glass slides with a drop of nail polish, ensuring that the cells faced upward. Hydrophobic barriers were drawn on the slides in the region surrounding the cells using the ImmEdge Hydrophobic Barrier Pen (Advanced Cell Diagnostics).

Cells were permeabilized in 0.1% (*v*/*v*) Tween 20 in PBS for 10 min before immunostaining or RNAscope. Immunostaining was performed using 10 μg/ml rabbit anti-human CD61 (Invitrogen, #MA5-32077), 10 μg/ml mouse IgG1 anti-human CD61 (Abcam, #ab218435), 10 μg/ml rabbit anti-human vWF (Abcam, #ab6994), 1:100 (*v*/*v*) rabbit IFTIM3 antibody (MA5-32798, Invitrogen), and 1:1000 (*v*/*v*) mouse IgG2b anti-HIV-1 p24 Gag Monoclonal (#24-4) (NIH AIDS Reagent Program, #ARP-6521) as primary antibodies (overnight incubation at 4°C) and 1:200 (*v*/*v*) of anti-mouse IgG1 AF488 (Jackson, #115-545-205), anti-mouse IgG2b AF488 (Jackson, #115-545-207), anti-rabbit AF488 (Invitrogen, #A21206), and anti-rabbit Cy5 (Jackson, #711-177-003) as secondary antibodies (2 h incubation) as previously described (Real et al., 2022). RNAscope was performed using the RNAscope Multiplex Fluorescent V2 Assay Kit (Advanced Cell Diagnostics) by incubating fixed and permeabilized cells with protease III for 10 min at RT and then *in situ* hybridizing these cells with the HIV Gag-Pol RNA probe (Advanced cell Diagnostics, #317691) for 2 h at 40°C. Next, hybridization was then amplified by incubating cells with Amp1, Amp2, Amp3, and HRP-C1 reagents (Advanced Cell Diagnostics) for 30, 30, 15, and 15 min, respectively. The cell nuclei were labeled with 10 μM 4′,6-diamidino-2-phenylindole (DAPI). Finally, coverslips were mounted with Mowiol 4–88 (Calbiochem) on glass slides.

Cells were analyzed using a Spinning disk unit (IXplore, Olympus) or LSM880 confocal laser-scanning microscope (Zeiss) equipped with 405, 488, 561, and 640 nm lasers and 60 ×/1.42 NA and 100 ×/1.4 oil objectives for detecting intracellular viral proteins/RNA. Acquired Z-stacks (Z-steps = 0.22 μm) were reconstructed into three-dimensional xy/xz/yz image projections and visualized using the Imaris software (version 10.0.2, Oxford Instruments).

### RT-qPCR and Alu-Gag PCR

After transfection of MEG-01 cells with siIFITM3 pools, total RNA was extracted from 5 × 10^5^ to 10^6^ cells using RNeasy MinElute Cleanup Kit (74204, Qiagen) at 24 h after transfection following the manufacturer's instructions. Samples with an appropriate RNA purity (A260/280 ≥ 1.8, A230/A260 ≥ 2.0) were selected. Viral RNA copy numbers were quantified by qPCR (TaqMan Gene Expression Assay, MGB-FAM-dye, Thermo Fisher Scientific) as described (Real et al., 2020, 2022) using the QuantStudio3 Thermocycler (Applied Biosystems). The selected primers RNase P/FW (5′-AGATTTGGACCTGCGAGCG-3′) and RNaseP/RV (5′-GAGCGGCTGTCTCCACAAGT-3′) (Eurogentec) were used together with the VIC-labeled TaqMan probe RNase P/Probe (VIC-TTCTGACCTGAAGGCTCTGCG-MGB) (Thermo Fisher Scientific).

The quantitative expression of IFITM3 was determined using the TaqMan Gene Expression Assay Hs03057129_s1 (Thermo Fisher Scientific). PCR was performed using the following thermocycler settings: 48°C for 15 min, 95°C for 10 min, 40 cycles of 95°C for 15 sec, and 60°C for 1 min using the QuantStudio3 Thermocycler (Applied Biosystems) in a total reaction volume of 20 μl. Each sample was analyzed in triplicate. For each sample, the CT (threshold cycle) for the RNA of interest was normalized to that for RNaseP. Fold induction was calculated by comparing normalized CT values (ΔΔCT) for two independent experiments. Relative gene expression data were calculated using the 2^−ΔΔ^*^C^*^t^ method (Livak and Schmittgen, 2001).

Alu-Gag PCR was performed as described (Real et al., 2020, 2022) including Gag-only controls (Liszewski et al., 2009) and peripheral mononuclear blood cell (PBMC) infected as described (Duchemin et al., 2020).

### Statistical analysis

Results were obtained from experiments repeated independently at least twice. The results are represented as dots for biological replicates (different HSPC donors) or independent experiments. Dot plots with more than two replicates also display the mean and SEM. Graphs were generated and statistical tests were performed using GraphPad Prism, with statistical tests being selected based on whether data were normally (parametric Student's *t*-test) or nonnormally distributed (nonparametric Mann–Whitney or Kruskal–Wallis tests), and significant differences were indicated by *P* < 0.05.

## Supplementary Material

mjae042_Supplemental_File
