# Supplementary material for: HIV-1 inhibits IFITM3 expression to promote the infection of megakaryocytes
Source: J Mol Cell Biol. 2024 Oct 1;16(9):mjae042. doi: 10.1093/jmcb/mjae042 (PMC11992561; doi:10.1093/jmcb/mjae042)
Supplement: mjae042_Supplemental_File [file mjae042_supplemental_file.pdf]

HIV-1 inhibits IFITM3 expression to promote its infection in megakaryocytes

Supplementary File

Supplementary Material and methods

Ethics statement

The use of human samples in this study was performed in accordance with the Declaration of Helsinki and Institutional Review Boards or other regulatory authority-approved consent forms and protocols. Primary cells were collected from healthy donor samples obtained from Établissement français du sang (EFS, France) and from a cord blood bank (STEMCELL and Lymphobank, Besançon, France) from which these cells were commercially available.

Gene expression analysis from public datasets

The public microarray whole transcriptome analysis dataset GSE77439 from the Gene Expression Omnibus (GEO) was reassessed using the GEO2R tool provided by the GEO website. This dataset includes the gene expression profiles of HSCs, CMPs, and two megakaryocyte progenitors (MEPs and MegPs) derived from bone marrow aspirates (Gene Expression Omnibus GSE77439). Datasets were grouped according to these 4 cell profiles and the expression of *CD4, CXCR4,* and *CCR5* was compared using the GEO2R default options. Expression values for the 4 cell types and for each of these 3 targets as provided by GEO2R software (Profile Graph tab) represent the expression measurements extracted from the Value column of the original submitter-supplied sample microarray record (Agilent-039494 SurePrint G3 Human GE v2 8x60K Microarray 039381). Negative values represent downregulation after data normalization using GeneSpring GX software (Agilent Technologies). Expression values were plotted in Rstudio as colorimetric scales ranging from black-purple (low expression, negative values) to orange-yellow (high expression, positive values).

CXCR4-tropic HIV-1 virus production

Prior to transfection with a plasmid encoding CXCR4-tropic HIV-1 (pNL4-3), HEK-293T (Cat. CRL-3216, ATCC) cells were expanded in Dulbecco's modified Eagle's medium (DMEM, Thermo Fisher Scientific) supplemented with 10% heat-inactivated fetal calf serum. Cells were seeded at a density of 10^6 cells/ml to ensure that they were in the logarithmic growth phase on the day of transfection. The pNL4-3 plasmid (Cat. ARP-114, NIH HIV Reagent Program) was transfected into HEK-293T cells using TurboFect Transfection Reagent (ThermoFisher) following the manufacturer's recommendations with a DNA to transfection reagent ratio of 1 to 3. The virus-containing supernatant was harvested at 72 h post-transfection following culture at 37°C and 5% CO_2, clarified, and saved for p24 measurement and HIV-1 titer determination as recommended by the NIH HIV Reagent Program. Viral stocks were snap-frozen in 1 ml aliquots containing 10^4 infectious units/ng p24-Gag and kept at -80°C.

Cell lysis and immunoblotting

Cells were lysed using B1 buffer (50 mM Tris–HCl pH 7.8 containing 100 mM NaCl, 2 mM EDTA, 1% Triton-X100, 0.1% SDS) supplemented with 1 mM PMSF and 1 × protease inhibitors (cOmplete™ protease inhibitor cocktail, Roche). The lysates were then stored at −20°C in 1 × Laemmli buffer until further use. Subsequently, the samples were loaded onto a 15% SDS-PAGE gel and transferred onto a nitrocellulose membrane (Hybond-ECL, Amersham) (1.5 h, 100 V). The membrane was incubated in a blocking buffer (PBS supplemented with nonfat dried milk 5% w/v and Tween-20 0.2% v/v) for 1 h at room temperature with constant shaking. Samples were then probed with either 1:1000 v/v rabbit monoclonal anti-human IFTIM3 antibody (MA5-32798, Invitrogen), 1:1000 v/v mouse monoclonal anti-human IFITIM2 antibody (Proteintech, 66137-1-Ig), or 1:1000 v/v rabbit monoclonal anti-human β-actin antibody (13E5 mAb 4970, Cell Signaling), which served as a normalization control. The membrane was washed three times with a solution of PBS Tween-20 0.2% v/v, followed by incubation with an appropriate peroxidase-conjugated secondary antibody in the blocking buffer for 45 min at room temperature. After three additional washes, protein detection was performed via enhanced chemiluminescence analysis (ECL, Amersham) following the manufacturer's protocol.

Densitometry analyses of western immunoblot bands were performed in the ImageJ2 (Fiji, v2.14.0/1.54f) software using 8-bit chemiluminescence images. Briefly, images were loaded into ImageJ, and the actin and IFITM3 bands were demarcated as the first and second lanes in the ImageJ-Analyze-Gel tool. The ImageJ-Analyze-Gel-Plot lanes command was then used to retrieve the band areas for the actin and IFITM3 chemiluminescence signals. Silencing efficiency was retrieved by calculating the ratio between IFITM3 and actin band areas, followed by normalizing the ratio obtained in siNTC-treated samples to the ratio obtained in siIFITM3-treated samples.

ELISA

Virus production in megakaryocyte supernatants was quantified as previously described (Real et al., 2018) via p24-Gag HIV capsid protein ELISA assessed at indicated time points using the INNOTEST HIV Antigen MAb (Fujirebio) according to the manufacturer’s instructions.

Real, F., Sennepin, A., Ganor, Y., et al. (2018). Live Imaging of HIV-1 Transfer across T Cell Virological Synapse to Epithelial Cells that Promotes Stromal Macrophage Infection. Cell Rep *23*, 1794–1805.

Supplementary Figures and Legends

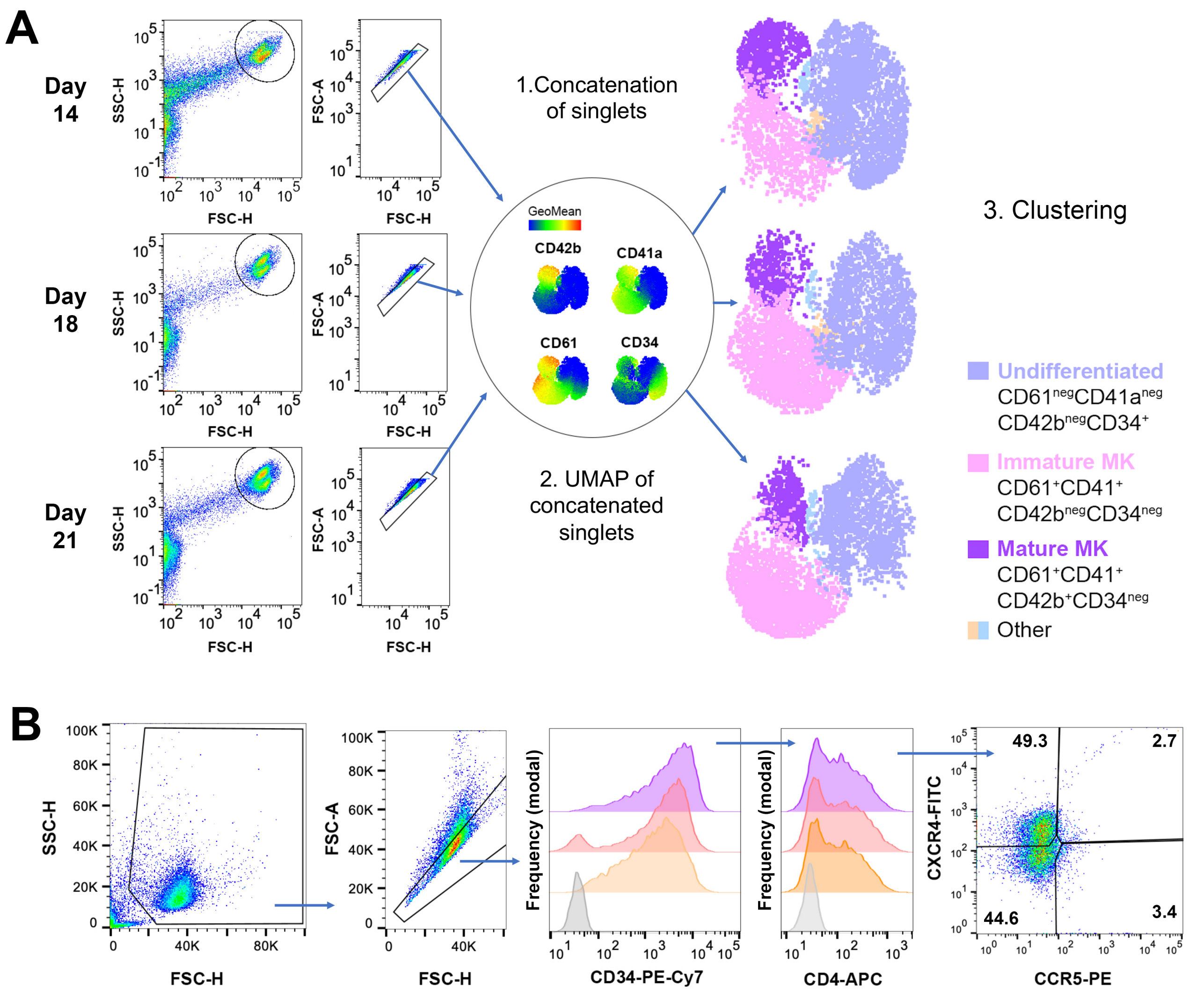

Supplementary Figure S1: (A) Flow cytometry gating strategy for profiling of HSPC-derived megakaryocytes during the differentiation protocol. Samples from 14, 18 and 21 days after start of differentiation were gated using FSC-H and SSC-H, followed by inclusion of singlets in an FSC-A/FSC-H dotplot. Next, singlets were concatenated and a UMAP was generated considering the expression of CD42b, CD41a, CD61 and CD34 expression. Clustering analysis (FlowSOM) allowed for visualizing undifferentiated cells, and differentiated megakaryocytes, separated in immature and mature populations. (B) Flow cytometry gating strategy for profiling CD4, CXCR4 and CCR5 expression on HSPC ($CD34^+$) used for differentiation toward megakaryocytes. Samples were gated in FSC-H/SSC-H dotplot, followed by inclusion of singlets in an FSC-A/FSC-H dotplot. Next, $CD34^+$ cells were gated in histograms. $CD34^+$ were then gated for $CD4^+$ cells in histograms. Finally, the frequency of $CXCR4^+$ and $CCR5^+$ cells were quantified among the $CD34^+CD4^+$ cells (as exemplified by the numbers displayed in the dotplot).

A

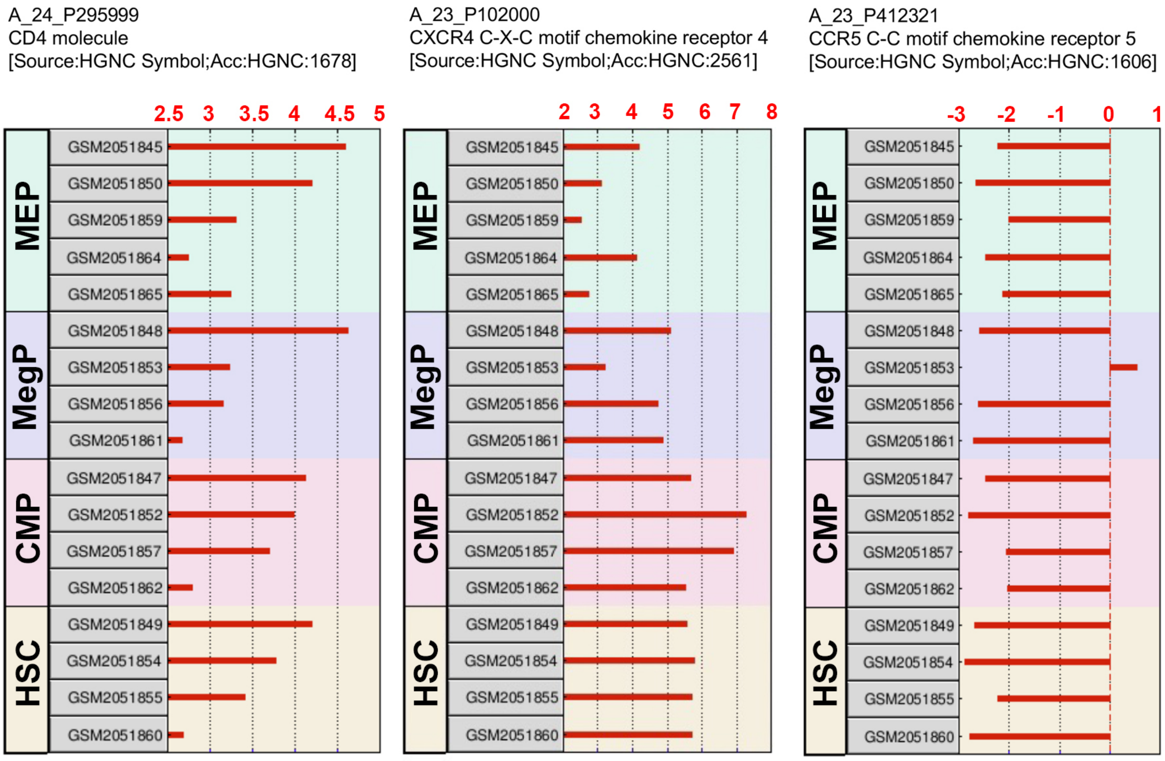

B

Sample	cell	replicate	*CD4* expression values	*CXCR4* expression values	*CCR5* expression values
GSM2051845	MEP	1	4.59805	4.22647	-2.23112
GSM2051850	MEP	2	4.21672	3.14748	-2.66993
GSM2051859	MEP	3	3.31566	2.55018	-2.03316
GSM2051864	MEP	4	2.76138	4.14708	-2.47118
GSM2051865	MEP	5	3.26206	2.76158	-2.13292
GSM2051848	MegP	1	4.62645	5.12158	-2.61041
GSM2051853	MegP	2	3.24085	3.22934	0.55004
GSM2051856	MegP	3	3.16425	4.75859	-2.63152
GSM2051861	MegP	4	2.68922	4.90659	-2.73277
GSM2051847	CMP	1	4.13101	5.71474	-2.47275
GSM2051852	CMP	2	4.00181	7.2955	-2.82468
GSM2051857	CMP	3	3.71721	6.92095	-2.07099
GSM2051862	CMP	4	2.81423	5.55914	-2.04643
GSM2051849	HSC	1	4.20399	5.59769	-2.7106
GSM2051854	HSC	2	3.7821	5.82448	-2.88031
GSM2051855	HSC	3	3.42454	5.7567	-2.23584
GSM2051860	HSC	4	2.70793	5.73157	-2.80643

Supplementary Figure S2: (A) Raw data obtained from Gene Expression Omnibus (GSE77439) after differentially expressed gene analysis performed comparing HSC, CMP, MEP and MegP cell profiles. CD4, CXCR4 and CCR5 were selected and the expression values given by the database as red bar graphs, per sequenced sample. Each red bar in the graph displays the expression values (x axis) of the gene across samples, representing the expression measurement extracted from the Value column of the original submitter-supplied sample microarray record (Agilent-039494 SurePrint G3 Human GE v2 8x60K Microarray 039381). Negative values represent downregulation after data normalization using GeneSpring GX software (Agilent Technologies). (B) Table of expression values containing numeric information.

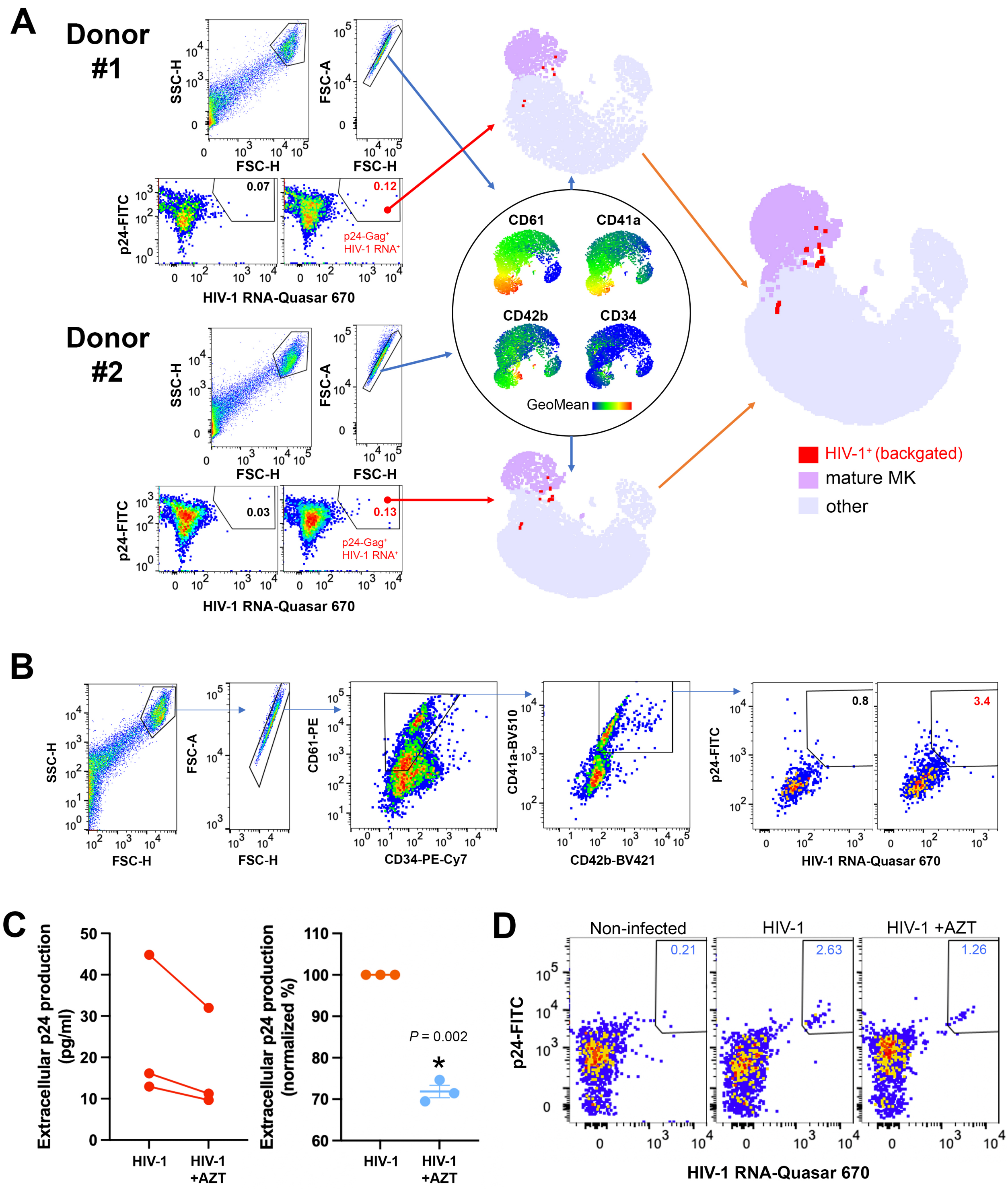

Supplementary Figure S3: (A) Flow cytometry gating strategy for profiling of identification of the cell profile containing HIV-1 p24-Gag and HIV-1 RNA (p24⁺/HIV-1 RNA⁺) among the cell population of HSPC-derived megakaryocytes. Samples from two different donors were gated using FSC-H and SSC-H, followed by exclusion of doublets in an FSC-A/FSC-H dotplot. Next, singlets were concatenated and a UMAP was generated considering the expression of CD42b, CD41a, CD61 and CD34 expression. Clustering analysis (FlowSOM) allowed for visualizing mature CD61⁺CD41a⁺CD42b⁺ megakaryocytes and other profiles (immature megakaryocytes or undifferentiated cells). p24⁺/HIV-1 RNA⁺ double-positive cells identified in each donor were backgated into UMAP of each donor (red dots in UMAPS). The frequencies of p24⁺/HIV-1 RNA⁺

double-positive cells in non-infected versus infected samples are displayed in the dotplots. Finally, the UMAPs and backgated events were combined into a single UMAP comprising results from each donor. (B) Flow cytometry gating strategy for assessing the frequency of mature megakaryocytes among total cells in culture and to assess infected $p24^+$/HIV-1 RNA^+ megakaryocytes among the mature megakaryocyte population. Samples were gated in FSC-H/SSC-H dotplot, followed by inclusion of singlets in an FSC-A/FSC-H dotplot. Next, $CD61^+CD34^{neg}$ cells were gated in dotplots, followed by gating $CD41a^+CD42b^+$ double-positive cells to assess mature megakaryocytes. Finally, the frequency of $p24^+$/HIV-1 RNA^+ double-positive cells were quantified among the mature megakaryocyte population infected or not by HIV-1 (exemplified by the numbers displayed in the dotplots). The frequency of infected cells was obtained by subtracting the background frequencies obtained in non-infected samples. (C) Extracellular p24 production detected in megakaryocyte cultures infected with HIV-1 and treated or not with AZT, expressed as pg/ml (left) and normalized by untreated cultures (right). Data refers to cumulative production at day 7 post-infection. Asterisk indicate statistical significance (Welch's T test, n=3 different cord blood donors). (D) FISH-flow quantification of the frequency of $p24^+$/HIV-1 RNA^+ cells in megakaryocyte preparations, infected with HIV-1 for 7 days, treated or not with AZT. Non-infected control is shown.

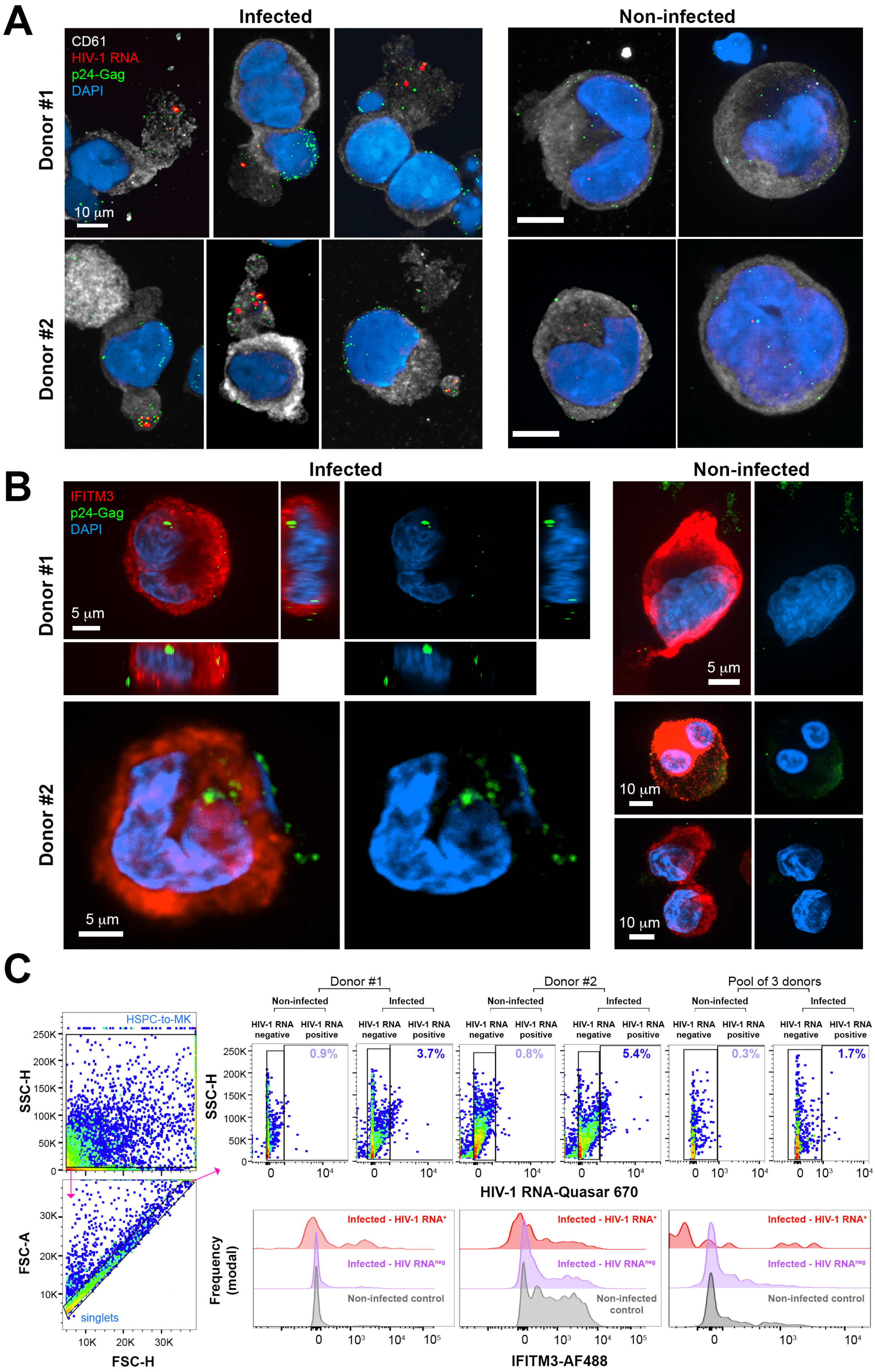

Supplementary Figure S4: (A) Confocal images of cord blood CD34+ cell-derived megakaryocytes infected

(upper row) or not (bottom) by HIV-1 NL4-3, immunostained by anti-p24-Gag (green) and anti-CD61 (white) antibodies and after HIV-1 RNA fluorescence *in situ* hybridization (red). Results comprise images obtained from 2 different cord blood donors. Nuclei stained by DAPI (blue). Bar = 5 or 10μm. (B) Confocal images of cord blood $CD34^+$ cell-derived megakaryocytes infected (left column) or not (right) by HIV-1 NL4-3, immunostained by anti-p24-Gag (green) and anti-IFITM3 (red) antibodies. Results comprise images obtained from 2 different cord blood donors (upper and bottom row). Nuclei stained by DAPI (blue). Bar = 5 or 10μm. (C) Gating strategy for FISH-flow coupled to IFITM3 staining in HSPC-derived megakaryocyte cultures (HSPC-to-MK). Cell population was gated using FSC-H and SSC-H, followed by exclusion of doublets in an FSC-A/FSC-H dotplot. Next, singlets were analyzed to gate HIV-1 RNA^+ and HIV-1 RNA^{neg} cell populations in non-infected and HIV-1-infected cell cultures (7 days post-infection). IFITM3 expression (GeoMean MFI) was analyzed in histograms for the gated populations of non-infected cells (gray: gated HIV-1 RNA^{neg}) and infected cells (purple: gated HIV-1 RNA^{neg} ; red: gated HIV-1 RNA^+). Results are representative of two independent experiments, one analyzing HSPC cultures from two different donors individually and the other analyzing HSPC cultures from a pool of three individuals.

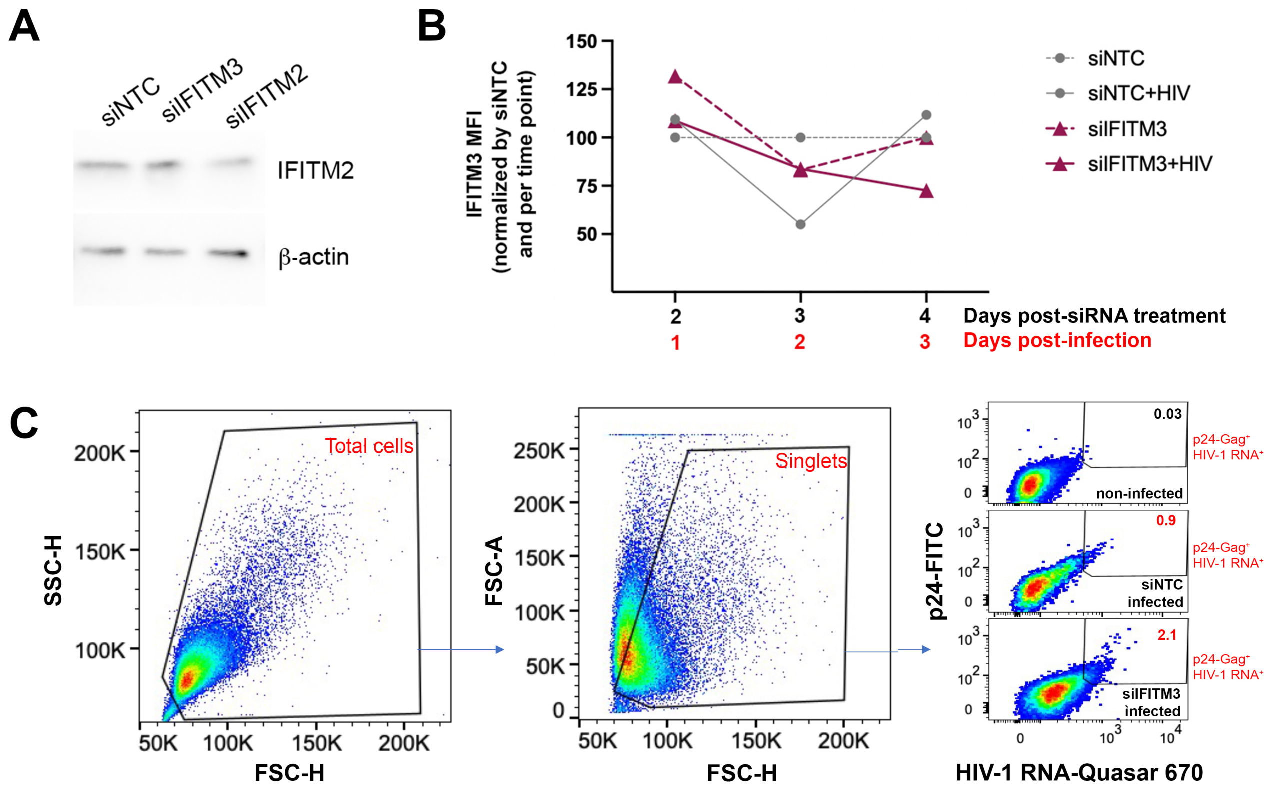

Supplementary Figure S5: (A) Western blot for IFITM2 in MEG-01 cells treated with non-targeting siRNA (siNTC), siIFITM3 and siIFITM2. Protein lysates were probed with an anti-IFITM2 antibody. Beta-actin immunodetection was used to normalize IFITM2 expression. (B) IFITM3 protein expression detected by FACS at different time points post-siRNA treatment, in MEG-01 cells infected or not by HIV-1, treated with siRNA non-targeting control (siNTC) or siIFITM3. Data were normalized by expression assessed in siNTC samples at each time point. HIV-1 infection was performed 24 h after siRNA treatment (days post-siRNA treatment and corresponding days post-infection are indicated in black and red, respectively. (C) Flow cytometry gating strategy for assessing the frequency of p24^{+}/HIV-1 RNA^{+} double-positive events among MEG-01 infected or not by HIV-1, transfected with siIFITM3 or siNTC. Samples were gated using FSC-H and SSC-H, followed by inclusion of singlets in a FSC-A/FSC-H dotplot. Next, frequency of HIV-1 p24^{+}/HIV-1 RNA^{+} double-positive events was retrieved (exemplified by the numbers displayed in the dotplots) and the frequency of infected cells were obtained by subtracting the background frequencies obtained in non-infected samples.
